# The Differences in the Pattern of OCT and OCTA Examinations between Early Normal- and High-Tension Pseudoexfoliative Glaucoma

**DOI:** 10.3390/jcm12154899

**Published:** 2023-07-26

**Authors:** Urszula Łukasik, Dominika Wróbel-Dudzińska, Jaromir Jarecki, Karolina Gasińska, Tomasz Żarnowski, Anna Święch, Ewa Kosior-Jarecka

**Affiliations:** 1 Department of Diagnostics and Microsurgery of Glaucoma, Medical University of Lublin, Chmielna Str. 1, 20-079 Lublin, Poland; urszula.lukasik@wp.pl (U.Ł.); doolores@interia.pl (D.W.-D.); karolina.gasinska@onet.eu (K.G.); tomaszzarnowski@umlub.pl (T.Ż.); 2 Department of Rehabilitation and Orthopaedics, Medical University of Lublin, 20-079 Lublin, Poland; 3 Department of Vitreoretinal Surgery, Medical University of Lublin, 20-079 Lublin, Poland; anna.zubi@umlub.pl

**Keywords:** OCT angiography, glaucoma, normal-tension glaucoma, high-tension glaucoma, early glaucoma, pseudoexfoliative glaucoma

## Abstract

**Purpose.** The aim of this study was to compare the results of optical coherence tomography angiography (OCTA) and optical coherence tomography (OCT) examinations in patients with normal-tension glaucoma (NTG) in comparison to high-tension pseudoexfoliative glaucoma (HTG) patients at the early stage of glaucoma. **Material and methods.** The studied groups consisted of patients in the early stage of NTG (70 eyes) and the early stage of HTG (71 eyes). In NTG and HTG groups, a detailed ophthalmic examination was performed. Optic disc OCT with peripapillary RNFL measurements and OCTA examination with the evaluation of the macula and optic disc were performed for all participants using Zeiss Cirrus 5000. **Results.** NTG and HTG groups were statistically similar as far as the MD was concerned, and both groups had early glaucoma. When evaluating the RNFL thickness, the only statistical difference between early NTG and HTG was observed in the thicknesses in the temporal sector of peripapillary RNFL, with thinner values in the NTG group (53.94 vs. 59.94, *p* = 0.0071). When the OCTA results of the macula and optic disc were evaluated, there were no statistical differences between early NTG and HTG. **Conclusions.** The vascular density and flow parameters assessed in OCTA were equal between early NTG and HTG, and therefore the involvement of vascular factors in NTG pathogenesis could not be confirmed. Our results confirm the preponderance of more frequent temporal RNFL involvement in early NTG.

## 1. Introduction

Glaucoma is a group of progressive optic neuropathies characterized by the degeneration of retinal ganglion cells and results in changes in the optic nerve head and the deterioration of the visual field, progressing from the periphery to the center, ultimately leading to irreversible blindness.

The major risk factors for glaucoma are elevated intraocular pressure (IOP) and advanced age. Elevated IOP is the only modifiable risk factor and all current therapies rely on its decrease using pharmaceutical or surgical methods [1]. However, there is now a growing body of evidence suggesting that multiple non-IOP factors can also cause glaucomatous optic neuropathy. Depending on the presence of elevated IOP at diagnosis, open-angle glaucoma is divided into high-tension glaucoma (HTG) and normal-tension glaucoma (NTG). Such division puts emphasis on different causative mechanisms involved in the pathogenesis of both types: increased IOP in HTG and other non-IOP-related mechanisms in NTG [2]. The possible involvement of different mechanisms is easiest to observe in the early stages of the disease.

Clinically, NTG is a subtype of primary open-angle glaucoma (POAG). Some studies report its frequency as high as 52–92% of all glaucoma cases, depending on the population [3]. Several authors confirmed that insufficient blood supply leads to retinal ganglion cell loss [4]. Low systemic blood pressure, especially with nocturnal decreases, has been reported to be more frequent in NTG patients [5,6], especially in progressive cases [7]. Pseudoexfoliative glaucoma is, on the other hand, a typical type of HTG, with a maximal IOP value even higher than that observed in POAG. The mechanism causing the increase in IOP is related to the accumulation of pseudoexfoliation debris on trabecular meshwork and an increase in the resistance of aqueous humor outflow.

Studies directly evaluating small ocular vasculature have not been previously possible, and the results of applied techniques have been elusive. Ocular blood flow monitoring never gained clinical relevance due to its lack of feasibility, high interindividual variability, and a lack of reproducible quantitative measurement methods. This trend changed with the development of optical coherence tomography angiography (OCTA), which allows for a quantitative and objective, dye-free, and non-invasive method to measure blood flow at the optic nerve head. It has been reported as a useful tool for evaluating optic disc perfusion in glaucomatous eyes since attenuated peripapillary and macular vessel density were detectable in preperimetric glaucoma patients [8]. However, little is known about the possible involvement of local vascular mechanisms observed in OCTA at the early stages of glaucoma.

The goal of this study was to evaluate the results of OCTA examination among patients with early visual field defects in two different types of glaucoma: NTG and pseudoexfoliative HTG. Additionally, we aimed to compare the RNFL measurements in OCT in these groups.

## 2. Material and Methods

The studied group consisted of 141 patients with early open-angle glaucoma treated in the Department of Diagnostics and Microsurgery of Glaucoma, the Medical University of Lublin, Poland. Written consent was obtained from all patients before their enrollment in the study, and the consent form was included in each patient’s study documentation. The study adhered to the tenets of the Declaration of Helsinki, and the study design was approved by the Bioethics Committee of the Medical University of Lublin (approval number: 127/21).

Open-angle glaucoma was diagnosed with the presence of glaucomatous neuroretinal rim loss, glaucomatous visual field (VF) damage in at least three valuable perimetric tests, and an open angle in gonioscopy. The early stage of glaucoma was diagnosed when the value of mean defect (MD) in any VF tests did not exceed −6 dB, based on the Hodapp-Parish–Anderson classification. Only one eye of each patient was included in the study. If both eyes were eligible, the eye with earlier damage was included according to MD in the VF examination. If the glaucomatous damage in both eyes was equal, the right eye was included.

The exclusion criteria were as follows: age less than 18 years old, refractive error worse than −6.0 D or higher than +3.0 D spherical equivalent, any pathology visible in the macula, diabetes mellitus, cataract with the best-corrected visual acuity (BVCA) less than 0.5, any visible retinopathy, history of trauma or uveitis, and inability to obtain reliable VF or optical coherence tomography (OCT) results. Patients with prior history of optic neuropathy or neurological disease or refractive surgery were not included in the study.

According to the value of the initial IOP, the 141 included eyes were divided into two groups: NTG patients and HTG patients. Early NTG was diagnosed if the untreated IOP obtained three times during office hours did not exceed the limit of 21 mmHg. The group of NTG patients comprised 70 eyes. For the HTG group, pseudoexfoliative glaucoma (PEXG) patients were recruited. The diagnosis of pseudoexfoliation syndrome was based on the presence of dandruff-like exfoliative material on the anterior lens capsule in the central disc and peripheral band (double concentric ring) pattern and/or in the anterior segment of the eye. In the case of pseudophakic eyes without any detected PEX material on slit-lamp examination, the diagnosis was based on medical records. All the included patients from the PEXG group had HTG. The PEXG group comprised 71 eyes. The IOP values were not corrected according to central corneal thickness (CCT). However, in the process of inclusion, when IOP correction was obtained and was higher than 21 mmHg in NTG cases or lower than 21 mmHg in PEXG eyes, the patients were excluded to avoid possible bias. The healthy gender- and age-matched control was formed from 75 patients without ophthalmic disorders excluding primary early senile cataracts. The epidemiologic characteristics of the studied groups are presented in Table 1.

After inclusion, the following parameters were assessed: the BCVA, using Snellen charts with decimal scale; slit lamp biomicroscopy with the evaluation of the anterior segment of the eye; gonioscopy, using Zeiss four-mirror gonioscope; as well as the stereoscopic fundus examination of the eye with a detailed assessment of the optic disc morphology. IOP was measured using Goldman applanation tonometry.

VF examinations were performed using a Humphrey Field analyzer II, model 720i (Zeiss Humphrey Systems, Dublin, CA, USA), with the Swedish interactive threshold algorithm 24-2 SITA FAST program. VFs were considered reliable if the fixation losses and false-negative response rates were ≤20% and the false-positive response rates were ≤15%.

SD-OCT and OCTA imaging were performed with Cirrus HD-OCT 5000 (Carl Zeiss Meditec Inc., Berlin, Germany). Only reliable OCT scans with a scan quality rate better than 5 were included in the analysis. Peripapillary retinal nerve fiber layer (RNFL) thickness scanning was performed using the optic disc cube 200 × 200 procedure. The thickness of the RNFL was calculated using the ONH and RNFL OU Analysis mode. All patients were additionally scanned using the AngioPlex OCTA system (Zeiss Meditec Inc., Dublin, CA, USA) covering a 6 × 6 mm scanning area centered at the optic nerve head (ONH) and covering a 6 × 6 mm and 3 × 3 mm scanning area centered at the foveola. The superficial layer of the optic disc area and the macular area were evaluated. The analysis of OCTA was performed including the following OCTA parameters: macular (foveal avascular zone (FAZ), perfusion density (PVD), and vessel density (VD)) and optic nerve head (optic nerve head perfusion and capillary flux index).

## 3. Results

NTG and HTG groups were statistically similar as far as the MD is concerned, and both groups had early glaucoma. When evaluating the RNFL thickness, the only statistical difference between early NTG and HTG was observed in the thicknesses in the temporal sector of peripapillary RNFL, with thinner values in the NTG group (53.94 vs. 59.94, *p* = 0.0071). The details of the RNFL comparison are provided in Table 2.

When the OCTA results of the macula were evaluated, there were no statistical differences in VD, PD, or FAZ parameters between early NTG and HTG considering both 6 × 6 and 3 × 3 scanning procedures. The detailed analysis results of these parameters in macular 6 × 6 scanning are presented in Table 3. The details of macular OCTA analysis results in 3 × 3 scanning are listed in Table 4.

When optic disc parameters were compared between early glaucoma groups, no statistical difference was observed for perfusion or flux measurements. The details of the analysis are listed in Table 5.

## 4. Discussion

Elevated IOP is the main causative factor in the pathogenesis of glaucoma. From the pathophysiological point of view, early damage to optic nerve fibers occurs at the level of lamina cribrosa, where the increased pressure gradient causes stress and strain. It results in compression, deformation, and remodeling of the lamina cribrosa and impedes neuronal transport within the axons forming the optic nerve [9,10,11]. However, glaucoma may also appear in cases when IOP never exceeds the statistical norm, and glaucomatous damage may develop in patients with an IOP value of low teens, which usually stabilizes the disease. There is a discussion on whether NTG is an undistinguishable form of POAG or a separate form of glaucoma with some distinct characteristics at the clinical, genetic, and therapeutic levels.

The glaucomatous damage developing in patients with normal IOP is explained by different theories. The historical term “normal-tension glaucoma” focuses on the absence of elevated IOP [2,12]. However, there are theories focusing on the other possible causative factors. Vascular failure, including vasospasms, small vessel disease, or autoregulatory dysfunction, is claimed to lead to perfusion deficits of the optic nerve head, retina, and choroid and, as a result, contributes to glaucoma development [13]. Previous studies showed that impaired vascular autoregulation was more pronounced in NTG than in HTG, especially in progressive cases [14,15]. The decreased blood flow was shown in NTG patients, compared with those with HTG in posterior bulbar circulation [16], as far as in nailfold capillaroscopy reflecting microcirculation [17,18], with reduced resting blood flow [19]. Additionally, Park et al. showed that the excessive vasospasm observed in the fingers of NTG patients is related to the progression of glaucoma despite very low IOP [20].

Previous studies reported attenuations in retinal microvasculature in POAG [21] and NTG [22] eyes, compared with healthy ones. Glaucoma severity seems to directly influence the strength of association between macular inner segments’ thickness and vascular density [8]. Hence, it remains unclear if the changes observed in microcirculation are the cause or the result of glaucoma. The studies of preperimetric/early glaucoma showed that GCC loss was greater than the macula vessel density loss [23]. On the other hand, significant microvascular damage was present in peripapillary areas [21]. The data comparing NTG and HTG are conflicting, and some of them show differences between these two types of glaucoma [24,25,26,27]. Shen et al. [28] compared the early stage of NTG and PAC; however, in their group of patients, the mean IOP values in both groups were similar.

In this study, we decided to compare early glaucoma patients with normal and high IOP. In the peripapillary RNFL assessment, we observed similar values in both groups in mean thicknesses, but the RNFL thickness was significantly lower in the temporal quadrant in NTG, with no other quadrant differences. In early glaucoma, RNFL defects are most frequently localized in the inferotemporal region, followed by the superotemporal region. Initially, glaucomatous VF defects usually involve the Bjerrum region with a pattern of arcuate-shaped scotoma sparing the paracentral area. However, a group of patients may exhibit RNFL damage close to the central region in the retina and the temporal region of the optic disc and present with paracentral or central scotomas in the VF [29]. Additionally, recent studies have reported early macular involvement within the course of glaucoma with central and paracentral VF defects. In our study, the significantly lower thickness of temporal RNFL in NTG patients compared with HTG patients confirms that this type of defect is more typical for patients with maximal IOP never exceeding the normal range [30]. Some studies showed that paracentral scotomas are related to systemic risk factors such as migraines, low systemic blood pressure, and other circulatory dysfunction [31,32,33]. Moreover, the incidences of color vision deficiency and decreased BCVA were reported to be more frequent in NTG patients with temporal RNFL defects than in NTG patients with inferotemporal or superotemporal RNFL defects [34]. An interesting hypothesis on the etiology of NTG patients with temporal RNFL defects is focused on the similarities between ophthalmic neuropathies with temporal RNFL defects and their association with mitochondrial dysfunction [34]. Interestingly, several studies have suggested that alterations in mitochondrial function may be related to glaucoma [34,35,36,37,38,39].

One of the surprising findings in our study is that the VD and PD of the macular region were all higher in the glaucoma group (NTG or HTG) than in the control group. In contrast, peripapillary OCTA parameters were lower in the glaucoma group than in the control group. There are two hypotheses that can explain this fact: the first points to possible compensatory mechanisms during early glaucoma, while the second indicates ineffective OCTA techniques.

Additionally, the control group had significantly higher RNFL thickness than both glaucomatous groups in all quadrants. However, OCTA parameters were not statistically different between the control and glaucomatous groups in the nasal quadrant. In the temporal quadrant, the difference was also only observed in the NTG group but not the PEX group. This may suggest that vascular changes follow the RNFL pattern of involvement during glaucoma, as RNFL nasal quadrants are the last affected and may be normal in early glaucoma.

The other surprising finding in this study is the lack of any differences in the OCTA of the macula and the optic disc in early NTG and HTG. We precluded that the vascular factors underlying NTG pathogenesis could be revealed in the OCTA small-vessel pattern. Our results may have some possible explanations. First, these results may indicate that the causative factors in at least a group of NTG patients are different from vascular factors [34], e.g., mitochondrial dysfunction. Moreover, this indicates that the NTG group may not form a united entity, and the clinical presentation of the disease is a better factor than the IOP level, allowing for the division of glaucoma into subgroups. The vascular changes contributing to glaucoma may also be related to the functional changes not visible at the morphological level. One of the risk factors of NTG is low general blood pressure or its fluctuations [5,7], which may not lead to specific capillary changes different from the primary vascular pathology.

Additionally, the other explanation is the complexity of the vascular blood supplementation of the optic nerve head and the retina. The studies show that the short posterior ciliary arteries in particular supply the choroid and optic nerve head and are vulnerable to changes in systemic blood pressure, perfusion pressure, and vascular dysregulation [40]. In OCTA, a small vessel, especially in retinal scanning, indicates the central retinal artery status. The retinal macular region is only supplied by the retinal artery, while the optic disc is supplied by the ciliary and retinal arteries. Moreover, the shape of peripapillary microvascular dropout in glaucoma reflects to a wedge-shaped glaucomatous RNFL defect, which shows that the changes observed in OCTA rather indicate a secondary process, not related to the primary cause of the pathology. The studies showed that retinal circulation was impaired only when IOP was elevated to the central retinal artery pressure. When IOP increased high enough, as occurs in primary angle closure glaucoma, the disc and peripapillary choroidal capillaries were obliterated, and the retinal circulation was slowed [41,42].

In this study, HTG patients with PEXG were recruited. The studied groups were chosen to maximally diversify the groups in terms of the level of IOP. Our PEXG patients were clearly HTG [43], with the mean IOP near 30 mmHg, higher than typical POAG and almost twice higher than our NTG group. However, some researchers claim that vascular disturbances are also involved in the pathogenesis of PEXG. In a previous meta-analysis, Wang et al. [44] showed that PEX increases the risk of vascular disease, coronary heart disease, cerebrovascular disease, and aortic aneurysm compared with the control group. Moreover, the narrowing of the small vessels in the PEX eye in a unilateral PEX was observed in some studies [45,46]. Yuksel et al. [47] found a significantly thinner RNFL in the PEX eye in a unilateral PEX without glaucoma, which suggested that an ocular blood flow disturbance through PEX deposition might contribute to the development of inner retinal atrophy. Hence, despite the different mechanisms of vascular disturbances in PEXG and NTG, it cannot be excluded that they may similarly influence the OCTA pattern. On the other hand, some studies have revealed that, although VD was reduced in the peripapillary and macular areas in POAG eyes, compared with the control [48,49], PEXG and POAG did not significantly differ in the disc and macular VD parameters [33], which is similar to our results for PEXG and NTG. Some studies also revealed that peripapillary VD was lower in eyes with PEXG than in eyes with POAG of similar severity [50] and pointed to choroidal microvascular dropout in PEXG [51]. However, in contrast to our study, they were not focused on the early stage of glaucoma. Comparisons of macular OCTA scans revealed no differences between POAG and NTG, but the glaucoma stage was not an inclusion criterion [26,52].

For the evaluation of systemic microcirculation, nailfold capillaries provide an accessible vascular bed visualization [53]. Additionally, nailfold capillaries exhibit morphologic features similar to ophthalmic vascular beds: The closed vascular loops with hairpin turns in the nailfold capillaries resemble the hairpin turns of vessels at the junction of the ONH and retina [54,55]. When comparing the nail-bed capillaroscopy between PEXG and NTG, the patterns of microvascular changes were slightly different for both diseases. In nail-bed capillaries of NTG patients, the presence of any dilated capillaries, avascular zones, and hemorrhages was noted [17,55,56], whereas in PEXG patients, the tortuosity of the capillaries was predominant but accompanied by avascular zones [57,58]. Additionally, in a previous study directly comparing the results of nail-bed videocapillaroscopy between PEXG and NTG, decreased resting peripheral capillary blood flow was observed in both groups of glaucoma patients, compared with individuals without glaucoma [59]. There are scarce studies that aim to translate the results of these two techniques by evaluating microvascular beds. The study by Shoji et al. [60] evaluated microvascular beds in the ONH, peripapillary tissue, and the nailfold in POAG patients versus controls and demonstrated concomitant abnormalities in ophthalmic microvasculature and nailfold capillaries in POAG with reduced vessel density and flow in the ophthalmic and systemic microvasculature. However, the significance of the differences in the microcapillary pattern between nail-bed capillaroscopy and OCTA needs further investigation.

And finally, OCTA may not be an adequate technique to study vascular patterns in glaucoma. OCTA undoubtedly has plenty of advantages, with high repeatability and reproducibility and good discriminatory power to differentiate normal eyes from glaucoma eyes. It is more strongly correlated with visual function than conventional OCT, reaches a floor effect at a more advanced stage of the disease, and is able to detect progression in glaucoma eyes [61]. There are doubts on whether the OCTA could be used to detect early glaucoma better than routine measurements of RNFL in the peripapillary region or GCC in the macular region. VD showed a more pronounced decrease as the severity of glaucoma increased. There are also studies showing worse intravisit repeatability and intervisit reproducibility of OCTA measurements, especially in glaucoma patients compared with healthy controls [62].

The difference in gender between the studied groups reflects the difference in the epidemiology of the studied glaucoma subtypes in our population. As we have described previously, NTG is more frequent in females [32], in contrast to PEXG, which is more common in males [43]. However, some studies suggest that gender may influence the OCTA parameters [63].

To summarize, in this study comparing early NTG and HTG, we found no differences in the peripapillary and macular parameters obtained in OCTA, and therefore the participation of local retinal vascular factors in NTG pathogenesis could not be confirmed. Our results confirm the preponderance of more frequent temporal RNFL involvement in NTG.

## Figures and Tables

**Table 1:** Demographic and clinical characteristics of the studied groups.

	NTG	HTG	Control	*p*-Level NTG vs. HTG	*p*-Level NTG vs. Control	*p*-Level HTG vs. Control
**Number**	70	71	75	X		
**Age (years)**	71.15 ± 7.25	72.91 ± 12.34	71.56 ± 7.63	0.3334	0.9443	0.6578
**Gender**	56F; 14M	42F; 29M	46F; 29M	0.0122 *	0.0179	0.2350
**maxIOP (mmHg)**	17.10 ± 2.37	29.05 ± 5.43	18.43 ± 2.12	0.0001 *	0.7852	0.0001 *
**VFI (%)**	87.43 ± 13.48	92.62 ± 12.30	98.32 ± 2.01	0.0219 *	0.0000 *	0.0001 *
**MD (dB)**	−3.70 ± 2.09	−3.51 ± 1.82	−0.12 ± 1.64	0.5647	0.0000 *	0000 *
**BCVA**	0.76 ± 0.23	0.74 ± 0.17	0.81 ± 0.11	0.7641	0.8634	0.4598
**Spherical Equivalent (D)**	−0.74 ± 1.46	−0.58 ± 1.87	−0.51 ± 1.11	0.7443	0.6532	0.8941

NTG normal tension glaucoma, HTG high tension glaucoma, IOP intraocular pressure, VFI visual field index. MD mean deviation, BCVA best corrected visual acuity. * statistically significant.

**Table 2:** Comparison of RNFL parameters between studied groups.

PARAMETER (mm)	NTG	HTG	Control	*p*-Level NTG vs. HTG	*p*-Level NTG vs. Control	*p*-Level HTG vs. Control
**Mean RNFL**	74.15 ± 11.31	73.48 ± 12.73	93.28 ± 10.27	0.7489	0.0000 *	0.0000 *
**Superior RNFL**	91.05 ± 18.23	87.19 ± 18.74	112.89 ± 17.42	0.2342	0.0001 *	0.0000 *
**Inferior RNFL**	85.50 ± 21.17	85.32 ± 25.90	119.61 ± 23.27	0.9659	0.0000 *	0.0000 *
**Temporal RNFL**	53.94 ± 11.72	59.94 ± 13.35	66.88 ± 11.24	0.0071 *	0.0000 *	0.0005 *
**Nasal RNFL**	66.17 ± 10.93	63.36 ± 9.15	73.50 ± 10.31	0.1153	0.0000 *	0.0000 *

NTG, normal tension glaucoma; HTG, high tension glaucoma; RNFL, retinal nerve fiber layer. * statistically significant.

**Table 3:** Comparison of macular OCTA parameters in 6 × 6 scans between NTG and HTG.

PARAMETER	NTG	HTG	*p*-Level
**Central VD**	8.98 ± 4.34	8.78 ± 4.10	0.7748
**Inner-ring VD**	16.22 ± 3.63	16.08 ± 3.17	0.8151
**Outer-ring VD**	15.70 ± 3.33	16.05 ± 3.14	0.5223
**Whole en face VD**	15.62 ± 3.30	15.86 ± 3.02	0.6626
**Central PD**	20.06 ± 9.95	19.84 ± 10.27	0.8983
**Inner-ring PD**	38.00 ± 9.34	37.74 ± 8.74	0.3300
**Outer-ring PD**	39.15 ± 8.91	40.28 ± 8.46	0.4463
**Whole en face PD**	38.63 ± 8.73	39.39 ± 8.10	0.5965
**FAZ area**	0.37 ± 0.80	0.24 ± 0.19	0.2170
**FAZ perimeter**	2.15 ± 1.04	2.05 ± 1.14	0.6157
**FAZ circularity**	0.86 ± 1.36	1.05 ± 2.03	0.7787

NTG, normal tension glaucoma; HTG, high tension glaucoma; PD, perfusion density; VD, vessel density; FAZ, foveal avascular zone.

**Table 4:** Comparison of macular OCTA parameters in 3 × 3 scans in studied groups.

	NTG	HTG	Control	*p*-Level NTG vs. HTG	*p*-Level NTG vs. Control	*p*-Level HTG vs. Control
**Central VD**	9.18 ± 4.00	9.53 ± 4.16	7.38 ± 3.48	0.6100	0.0052 *	0.0030 *
**Inner-ring VD**	19.19 ± 3.70	18.85 ± 3.32	17.38 ± 3.17	0.5683	0.0041 *	0.0196 *
**Whole en face VD**	18.08 ± 3.47	17.81 ± 3.24	16.25 ± 3.23	0.6325	0.0023 *	0.0111 *
**Central PD**	16.80 ± 7.01	17.50 ± 7.66	13.06 ± 7.13	0.5759	0.0013 *	0.0008 *
**Inner-ring PD**	35.85 ± 6.48	35.10 ± 6.30	32.50 ± 6.12	0.4915	0.0023 *	0.0209 *
**Whole en face PD**	33.65 ± 6.00	33.50 ± 5.71	30.29 ± 5.34	0.8772	0.0013 *	0.0033 *
**FAZ Area**	0.26 ± 0.13	0.24 ± 0.11	0.25 ± 0.11	0.3383	0.3327	0.7998
**FAZ perimeter**	2.26 ± 0.85	2.18 ± 0.65	2.12 ± 0.73	0.5624	0.4015	0.4441
**Faz circularity**	0.60 ± 0.14	0.62 ± 0.09	0.61 ± 0.11	0.3334	0.3177	0.9111

NTG, normal tension glaucoma; HTG, high tension glaucoma; PD, perfusion density; VD, vessel density; FAZ, foveal avascular zone. * statistically significant.

**Table 5:** Comparison between OCTA parameters of the optic disc between NTG, HTG, and control groups.

	NTG	HTG	Control	*p*-Level NTG vs. HTG	*p*-Level NTG vs. Control	*p*-Level HTG vs. Control
	**Circumpapillary perfusion (in %)**					
**Average ONH**	42.90 ± 2.87	42.95 ± 3.39	44.66 ± 2.43	0.9354	0.0000 *	0.0001 *
**Superior**	41.43 ± 4.26	41.10 ± 6.44	42.74 ± 5.76	0.7301	0.0362 *	0.0216 *
**Inferior**	40.59 ± 4.48	40.23 ± 7.53	44.89 ± 4.83	0.7403	0.0000 *	0.0000 *
**Temporal**	45.57 ± 2.89	45.27 ± 4.10	47.07 ± 2.32	0.3216	0.0035 *	0.3060
**Nasal**	43.68 ± 3.32	43.35 ± 4.78	43.72 ± 2.67	0.6529	0.9981	0.3772
	**Circumpapillary flux (%)**					
**ONH Flux index**	0.37 ± 0.04	0.38 ± 0.05	0.41 ± 0.04	0.8432	0.0000 *	0.0000 *
**Superior flux**	0.37 ± 0.04	0.37 ± 0.05	0.40 ± 0.04	0.6548	0.0001 *	0.0001 *
**Inferior flux**	0.37 ± 0.04	0.37 ± 0.05	0.40 ± 0.05	0.3580	0.0000 *	0.0000 *
**Temporal flux**	0.39 ± 0.05	0.39 ± 0.05	0.42 ± 0.05	0.7800	0.0000 *	0.0001 *
**Nasal Flux**	0.37 ± 0.05	0.38 ± 0.05	0.40 ± 0.05	0.8049	0.0001 *	0.0002 *

NTG, normal tension glaucoma; HTG, high tension glaucoma; ONH, optic nerve head. * statistically significant.

## Data Availability

On request from corresponding author.

## References

[1] Nakazawa T., Fukuchi T. (2020). What is glaucomatous optic neuropathy?. Jpn. J. Ophthalmol..

[2] Killer H.E., Pircher A. (2018). Normal tension glaucoma: Review of current understanding and mechanisms of the pathogenesis. Eye.

[3] Cho H.K., Kee C. (2014). Population-based glaucoma prevalence studies in Asians. Surv. Ophthalmol..

[4] Yamazaki Y., Drance S.M. (1997). The relationship between progression of visual field defects and retrobulbar circulation in patients with glaucoma. Am. J. Ophthalmol..

[5] Hayreh S.S., Zimmerman M.B., Podhajsky P., Alward W.L. (1994). Nocturnal arterial hypotension and its role in optic nerve head and ocular ischemic disorders. Am. J. Ophthalmol..

[6] Kosior-Jarecka E., Łukasik U., Wróbel-Dudzińska D., Kocki J., Bartosińska J., Witczak A., Chodorowska G., Mosiewicz J., Żarnowski T. (2016). Risk Factors for Normal and High-Tension Glaucoma in Poland in Connection with Polymorphisms of the Endothelial Nitric Oxide Synthase Gene. PLoS ONE..

[7] Graham S.L., Drance S.M. (1999). Nocturnal hypotension: Role in glaucoma progression. Surv. Ophthalmol..

[8] Lever M., Glaser M., Chen Y., Halfwassen C., Unterlauft J.D., Bechrakis N.E., Böhm M.R.R. (2021). Microvascular and Structural Alterations of the Macula in Early to Moderate Glaucoma: An Optical Coherence Tomography-Angiography Study. J. Clin. Med..

[9] Lichter P.R., Musch D.C., Gillespie B.W., Guire K.E., Janz N.K., Wren P.A., Mills R.P., CIGTS Study Group (2001). Interim clinical outcomes in the Collaborative Initial Glaucoma Treatment Study comparing initial treatment randomized to medications or surgery. Ophthalmology.

[10] Anderson D.R., Drance S.M., Schulzer M., Collaborative Normal-Tension Glaucoma Study Group (2003). Factors that predict the benefit of lowering intraocular pressure in normal tension glaucoma. Am. J. Ophthalmol..

[11] Jonas J.B., Aung T., Bourne R.R., Bron A.M., Ritch R., Panda-Jonas S. (2017). Glaucoma. Lancet.

[12] Jonas J.B., Wang N. (2013). Cerebrospinal fluid pressure and glaucoma. J. Ophthalmic Vis. Res..

[13] Plange N., Remky A., Arend O. (2003). Colour Doppler imaging and fluorescein filling defects of the optic disc in normal tension glaucoma. Br. J. Ophthalmol..

[14] Kaiser H.J., Schoetzau A., Stümpfig D., Flammer J. (1997). Blood-flow velocities of the extraocular vessels in patients with high-tension and normal-tension primary open-angle glaucoma. Am. J. Ophthalmol..

[15] Grunwald J.E., Piltz J., Hariprasad S.M., DuPont J. (1998). Optic nerve and choroidal circulation in glaucoma. Investig. Ophthalmol. Vis. Sci..

[16] Barbosa-Breda J., Van Keer K., Abegão-Pinto L., Nassiri V., Molenberghs G., Willekens K., Vandewalle E., Rocha-Sousa A., Stalmans I. (2019). Improved discrimination between normal-tension and primary open-angle glaucoma with advanced vascular examinations—The Leuven Eye Study. Acta Ophthalmol..

[17] Kosior-Jarecka E., Bartosińska J., Łukasik U., Wróbel-Dudzińska D., Krasowska D., Chodorowska G., Żarnowski T. (2018). Results of Nailfold Capillaroscopy in Patients with Normal-Tension Glaucoma. Curr. Eye Res..

[18] Gasser P., Flammer J. (1991). Blood-cell velocity in the nailfold capillaries of patients with normal-tension and high-tension glaucoma. Am. J. Ophthalmol..

[19] Cousins C.C., Chou J.C., Greenstein S.H., Brauner S.C., Shen L., Turalba A.V., Houlihan P., Ritch R., Wiggs J.L., Knepper P.A. (2019). Resting nailfold capillary blood flow in primary open-angle glaucoma. Br. J. Ophthalmol..

[20] Park D.Y., Han J.C., Lee E.J., Kee C. (2021). Relationship between peripheral vasospasm and visual field progression rates in patients with normal-tension glaucoma with low-teen intraocular pressure. PLoS ONE.

[21] Lu P., Xiao H., Liang C., Xu Y., Ye D., Huang J. (2020). Quantitative Analysis of Microvasculature in Macular and Peripapillary Regions in Early Primary Open-Angle Glaucoma. Curr. Eye Res..

[22] Lin T.P.H., Wang Y.M., Ho K., Wong C.Y.K., Chan P.P., Wong M.O.M., Chan N.C.Y., Tang F., Lam A., Leung D.Y.L. (2020). Global assessment of arteriolar, venular and capillary changes in normal tension glaucoma. Sci. Rep..

[23] Hou H., Moghimi S., Zangwill L.M., Shoji T., Ghahari E., Penteado R.C., Akagi T., Manalastas P.I.C., Weinreb R.N. (2019). Macula Vessel Density and Thickness in Early Primary Open-Angle Glaucoma. Am. J. Ophthalmol..

[24] Park J.H., Yoo C., Kim Y.Y. (2019). Peripapillary Vessel Density in Young Patients with Open-Angle Glaucoma: Comparison between High-Tension and Normal-Tension Glaucoma. Sci. Rep..

[25] Scripsema N.K., Garcia P.M., Bavier R.D., Chui T.Y.P., Krawitz B.D., Mo S., Agemy S.A., Xu L., Lin Y.B., Panarelli J.F. (2016). Optical Coherence Tomography Angiography Analysis of Perfused Peripapillary Capillaries in Primary Open-Angle Glaucoma and Normal-Tension Glaucoma. Investig. Ophthalmol. Vis. Sci..

[26] Xu H., Zhai R., Zong Y., Kong X., Jiang C., Sun X., He Y., Li X. (2018). Comparison of retinal microvascular changes in eyes with high-tension glaucoma or normal-tension glaucoma: A quantitative optic coherence tomography angiographic study. Graefe’s Arch. Clin. Exp. Ophthalmol..

[27] Bojikian K.D., Chen C.L., Wen J.C., Zhang Q., Xin C., Gupta D., Mudumbai R.C., Johnstone M.A., Wang R.K., Chen P.P. (2016). Optic Disc Perfusion in Primary Open Angle and Normal Tension Glaucoma Eyes Using Optical Coherence Tomography-Based Microangiography. PLoS ONE.

[28] Shen R., Wang Y.M., Cheung C.Y., Chan P.P., Tham C.C. (2021). Comparison of optical coherence tomography angiography metrics in primary angle-closure glaucoma and normal-tension glaucoma. Sci. Rep..

[29] Leung C.K., Yu M., Weinreb R.N., Lai G., Xu G., Lam D.S. (2012). Retinal nerve fiber layer imaging with spectral-domain optical coherence tomography: Patterns of retinal nerve fiber layer progression. Ophthalmology.

[30] Yum H.R., Park H.L., Park C.K. (2020). Characteristics of Normal-tension Glaucoma Patients with Temporal Retinal Nerve Fibre Defects. Sci. Rep..

[31] Kosior-Jarecka E., Wróbel-Dudzińska D., Łukasik U., Żarnowski T. (2017). Ocular and Systemic Risk Factors of Different Morphologies of Scotoma in Patients with Normal-Tension Glaucoma. J. Ophthalmol..

[32] Kosior-Jarecka E., Wróbel-Dudzińska D., Łukasik U., Żarnowski T. (2019). Disc haemorrhages in Polish Caucasian patients with normal tension glaucoma. Acta Ophthalmol..

[33] Jo Y.H., Sung K.R., Shin J.W. (2020). Peripapillary and Macular Vessel Density Measurement by Optical Coherence Tomography Angiography in Pseudoexfoliation and Primary Open-angle Glaucoma. J. Glaucoma.

[34] Abu-Amero K.K., Morales J., Bosley T.M. (2006). Mitochondrial abnormalities in patients with primary open-angle glaucoma. Investig. Ophthalmol. Vis. Sci..

[35] Ju W.K., Kim K.Y., Lindsey J.D., Angert M., Duong-Polk K.X., Scott R.T., Kim J.J., Kukhmazov I., Ellisman M.H., Perkins G.A. (2008). Intraocular pressure elevation induces mitochondrial fission and triggers OPA1 release in glaucomatous optic nerve. Investig. Ophthalmol. Vis. Sci..

[36] Lee S., Sheck L., Crowston J.G., Van Bergen N.J., O’Neill E.C., O’Hare F., Kong Y.X.G., Chrysostomou V., Vincent A.L., Trounce I.A. (2012). Impaired complex-I-linked respiration and ATP synthesis in primary open-angle glaucoma patient lymphoblasts. Investig. Ophthalmol. Vis. Sci..

[37] Jeoung J.W., Seong M.W., Park S.S., Kim D.M., Kim S.H., Park K.H. (2014). Mitochondrial DNA variant discovery in normal-tension glaucoma patients by next-generation sequencing. Investig. Ophthalmol. Vis. Sci..

[38] Piotrowska-Nowak A., Kosior-Jarecka E., Schab A., Wrobel-Dudzinska D., Bartnik E., Zarnowski T., Tonska K. (2019). Investigation of whole mitochondrial genome variation in normal tension glaucoma. Exp. Eye Res..

[39] Milanowski P., Kosior-Jarecka E., Łukasik U., Wróbel-Dudzińska D., Milanowska J., Khor C.C., Aung T., Kocki J., Żarnowski T. (2022). Associations between *OPA1, MFN1*, and *MFN2* polymorphisms and primary open angle glaucoma in Polish participants of European ancestry. Ophthalmic Genet..

[40] Prada D., Harris A., Guidoboni G., Siesky B., Huang A.M., Arciero J. (2016). Autoregulation and neurovascular coupling in the optic nerve head. Surv. Ophthalmol..

[41] Rong X., Cai Y., Li M., Chen X., Kang L., Yang L. (2020). Relationship between nailfold capillary morphology and retinal thickness and retinal vessel density in primary open-angle and angle-closure glaucoma. Acta Ophthalmol..

[42] Zha Y., Chen J., Liu S., Zhuang J., Cai J. (2020). Vessel Density and Structural Measurements in Primary Angle-Closure Suspect Glaucoma Using Optical Coherence Tomography Angiography. Biomed Res. Int..

[43] Łukasik U., Kosior-Jarecka E., Wróbel-Dudzińska D., Kustra A., Milanowski P., Żarnowski T. (2020). Clinical Features of Pseudoexfoliative Glaucoma in Treated Polish Patients. Clin. Ophthalmol..

[44] Wang W., He M., Zhou M., Zhang X. (2014). Ocular pseudoexfoliation syndrome and vascular disease: A systematic review and meta-analysis. PLoS ONE.

[45] Takai Y., Tanito M., Omura T., Kawasaki R., Kawasaki Y., Ohira A. (2017). Comparisons of retinal vessel diameter and glaucomatous parameters between both eyes of subjects with clinically unilateral pseudoexfoliation syndrome. PLoS ONE.

[46] Oruc Y., Kirgiz A. (2019). Alteration of Retinal Vessel Diameter of the Patients with Pseudoexfoliation and Optical Coherence Tomography Images. Curr. Eye Res..

[47] Yüksel N., Altintaş O., Celik M., Ozkan B., Cağlar Y. (2007). Analysis of retinal nerve fiber layer thickness in patients with pseudoexfoliation syndrome using optical coherence tomography. Ophthalmologica.

[48] Yarmohammadi A., Zangwill L.M., Diniz-Filho A., Suh M.H., Manalastas P.I., Fatehee N., Yousefi S., Belghith A., Saunders L.J., Medeiros F.A. (2016). Optical Coherence Tomography Angiography Vessel Density in Healthy, Glaucoma Suspect, and Glaucoma Eyes. Investig. Ophthalmol. Vis. Sci..

[49] Rao H.L., Pradhan Z.S., Weinreb R.N., Reddy H.B., Riyazuddin M., Dasari S., Palakurthy M., Puttaiah N.K., Rao D.A., Webers C.A. (2016). Regional Comparisons of Optical Coherence Tomography Angiography Vessel Density in Primary Open-Angle Glaucoma. Am. J. Ophthalmol..

[50] Park J.H., Yoo C., Girard M.J.A., Mari J.M., Kim Y.Y. (2018). Peripapillary Vessel Density in Glaucomatous Eyes: Comparison Between Pseudoexfoliation Glaucoma and Primary Open-angle Glaucoma. J. Glaucoma.

[51] Pradhan Z.S., Rao H.L., Dixit S., Sreenivasaiah S., Reddy P.G., Venugopal J.P., Puttaiah N.K., Devi S., Weinreb R.N., Mansouri K. (2019). Choroidal Microvascular Dropout in Pseudoexfoliation Glaucoma. Investig. Ophthalmol. Vis. Sci..

[52] Mursch-Edlmayr A.S., Waser K., Podkowinski D., Bolz M. (2020). Differences in swept-source OCT angiography of the macular capillary network in high tension and normal tension glaucoma. Curr. Eye Res..

[53] Etehad Tavakol M., Fatemi A., Karbalaie A., Emrani Z., Erlandsson B.E. (2015). Nailfold Capillaroscopy in Rheumatic Diseases: Which Parameters Should Be Evaluated?. Biomed. Res. Int..

[54] Chandrasekera E., An D., McAllister I.L., Yu D.Y., Balaratnasingam C. (2018). Three-Dimensional Microscopy Demonstrates Series and Parallel Organization of Human Peripapillary Capillary Plexuses. Investig. Ophthalmol. Vis. Sci..

[55] Park H.Y., Park S.H., Oh Y.S., Park C.K. (2011). Nail bed hemorrhage: A clinical marker of optic disc hemorrhage in patients with glaucoma. Arch. Ophthalmol..

[56] Pasquale L.R., Hanyuda A., Ren A., Giovingo M., Greenstein S.H., Cousins C., Patrianakos T., Tanna A.P., Wanderling C., Norkett W. (2015). Nailfold Capillary Abnormalities in Primary Open-Angle Glaucoma: A Multisite Study. Investig. Ophthalmol. Vis. Sci..

[57] Cousins C.C., Kang J.H., Bovee C., Wang J., Greenstein S.H., Turalba A., Shen L.Q., Brauner S., Boumenna T., Blum S. (2017). Nailfold capillary morphology in exfoliation syndrome. Eye.

[58] Łukasik U., Bartosińska J., Kosior-Jarecka E., Wróbel-Dudzińska D., Krasowska D., Żarnowski T. (2023). Results of Nailfold Videocapillaroscopy in Patients with Pseudoexfoliative Glaucoma. Life.

[59] Philip S., Najafi A., Tantraworasin A., Pasquale L.R., Ritch R. (2019). Nailfold Capillaroscopy of Resting Peripheral Blood Flow in Exfoliation Glaucoma and Primary Open-Angle Glaucoma. JAMA Ophthalmol..

[60] Shoji M.K., Cousins C.C., Saini C., e Silva R.N., Wang M., Brauner S.C., Greenstein S.H., Pasquale L.R., Shen L.Q. (2021). Paired Optic Nerve Microvasculature and Nailfold Capillary Measurements in Primary Open-Angle Glaucoma. Transl. Vis. Sci. Technol..

[61] Van Melkebeke L., Barbosa-Breda J., Huygens M., Stalmans I. (2018). Optical Coherence Tomography Angiography in Glaucoma: A Review. Ophthalmic Res..

[62] Rao H.L., Pradhan Z.S., Suh M.H., Moghimi S., Mansouri K., Weinreb R.N. (2020). Optical Coherence Tomography Angiography in Glaucoma. J. Glaucoma.

[63] Bazvand F., Mirshahi R., Fadakar K., Faghihi H., Sabour S., Ghassemi F. (2017). The Quantitative Measurements of Vascular Density and Flow Area of Optic Nerve Head Using Optical Coherence Tomography Angiography. J. Glaucoma.

